# Correction: Garbuz et al. Design, Optimization and Validation of the ARMS PCR Protocol for the Rapid Diagnosis of Wilson’s Disease Using a Panel of 14 Common Mutations for the European Population. *Genes* 2022, *13*, 1940

**DOI:** 10.3390/genes15081070

**Published:** 2024-08-14

**Authors:** Mikhail Maksimovich Garbuz, Anna Alexandrovna Ovchinnikova, Vadim Vladimirovich Kumeiko

**Affiliations:** 1 Institute of Life Sciences and Biomedicine, School of Natural Sciences, Far Eastern Federal University, Vladivostok 690922, Russia; garbuzmihail.93@gmail.com (M.M.G.);; 2 A.V. Zhirmunsky National Scientific Center of Marine Biology, Far Eastern Branch of Russian Academy of Sciences, Federal University, Vladivostok 690041, Russia

In the original publication [1], the affiliation 1 was updated. There was also a mistake in Tables 1 and 2 as published. The errors were technical and occurred while transferring data into a table using a template. To avoid problems with reproducibility, and the possibility of our method being used by interested people, we request that these changes to be made to Tables 1 and 2. The corrected Table 1 and Table 2 appear below. The authors confirm that the scientific conclusions are unaffected. This correction was approved by the Academic Editor. The original publication has also been updated.

## Figures and Tables

**Table 1:** Primers for exon amplification of the *ATP7B* gene.

Exon	Forward Primer	Revers Primer
2A	GGCATTGTTTTCCATTTTCTCAGTG	CATGTCCCCAATTTGATGGCAAAC
2B	GAAGGTTTCCCTGGAACAAGGC	GCAGAAGATAAAGGTCTCTTTGGG
2C	TGGGACCAATTGATATTGAGCGG	GGAAGACCTGTGATCTGTCCC
2D	TATCGAGGCACTTCCACCTGG	CTCACCTATACCACCATCCAGG
3	GGTGGGAGCCGGGACAATGAACC	CAGCATTCCTAAGTTCAACATGGG
4	TATTGACTGTGTCAACCTAGAGGC	AAACTGTCAGAAGCCTGTAACCC
5	CTGCCTGTTACCTAGACTCCC	TTACCCATTCACTGATATCCTCCC
6	AAAACCCACAAAGTCTACTGAGGC	CAGCTAATCCAGGAGGAAGGC
7	TCTTAAACTGTGTCCTCAGAAGGG	ACTATGTTTGCGCTTAGCGGGC
8	GACTGTGCACAAAGCTAGAGGC	GAGATTTGTTTACTGAAGGAGCAGC
9	GTGTGGTGGATAGCAAGTAACGC	CTTTCGTAGCTGGATTGAGAGTGG
10 + 11	AGCTGGCCTAGAACCTGACCC	GAACTCTTCACATAATTTCTAAAACGAGA
12	CCCAATCTTTATCCATGCTTGTGG	AGTGACTGTTTATCCTACTCTGGC
13	CCTTATTGAACTCTCAACCTGCC	CTCTGTTGCTACTGTTGTTATTCCC
14	CCCTGAGATTGAACGACAGAGG	GGTGAGGAATAAAAGAGCATTGGC
15	CTTTCCGCTGCTCTCTTGCC	CAGAGGCAATCACTGCTGGG
16	TGTCACAAGAGGTGCTTACAAGG	GCCTGAAATTAAGAGAGGAAGGC
17	CTTCCAGACTTTTGTGTACATCCG	GAGTACAGCTCAGTGCTGGG
18 + 19	TTTTGCCAACACTAGGCATTGCC	GGAGACAGAAGCCTTTCTGGG
20	GAACATCAGGGCGAGTGGAAG	GTGCTAAGCATGCAGAATGACAAG

**Table 2:** Primers for allele-specific PCR of the *ATP7B* mutation sites.

Exon	Mutation	Mutant forward Primer	Normal forward Primer	Revers Primer
5	Arg616Gln	TTTGACCCGGAAATTATCGGTCCATA	GACCCGGAAATTATCGGTCCATG	CAAGCTCTCCACAACAAGAGTGG
8	Met769HisfsTer26	CATTCTTCGACACGCCCCCTC	ACATTCTTCGACACGCCCCCTA	TTTGGAGATTAGTGACTAGAGCACCT
	Gly710Ser	CTGTCCTTGTCTTTCAGCTCCTTA	CTGTCCTTGTCTTTCAGCTCCTTG	
	Ser744Pro	GGCCACAAGCATTGCTTATGTTTTTC	TGGCCACAAGCATTGCTTATGTTTTTT	
	Arg778Leu	CTTTGTGTTCATTGCCCTGGGTCT	TTTGTGTTCATTGCCCTGGGTCG	GACCAACTACATATTCAGTTTTGCACC
	Arg778Gly	CTTTGTGTTCATTGCCCTGGGTG	CTTTGTGTTCATTGCCCTGGGTC	
	Trp779*	TCATTGCCCTGGGCCGGTCA	TTCATTGCCCTGGGCCGGTTG	
10	Val834Asp	GGGGCGATATCGTCAAGGTGTA	GGGGCGATATCGTCAAGGTGTT	GTCTGATTTCCCAGAACTCTTCACATA
12	Gly943Ser	TTGACGTTGGTGGTATGGATTGTAATTA	GACGTTGGTGGTATGGATTGTAATTG	CAACTGAGCACCAATTGGTGTCTG
14	His1069Gln	TGCGGAGGCCAGCAGTGAATAA	GCGGAGGCCAGCAGTGAATAC	AGACTGCCCGTACTCCCCAAG
	Glu1064Lys	GGCTGTGGTGGGGACTGCTA	GCTGTGGTGGGGACTGCTG	
	Arg1041Trp	GGCGTCCCCAGGGTCACGT	GCGTCCCCAGGGTCACGC	
	3222_3243+21del43	GGCGTGGCGTACGTGGACTT	GTGGCAGTCACCAAATACTGTAAAG	
15	Ala1135GlnfsTer13	TGAGGCTGGCAGCCTTCCCTA	AGGCTGGCAGCCTTCCCTC	ACATTTCCCTCTGCTTTCCCTGAC
